# Rescue Emergencies Due to High-Altitude Illnesses Are Rare in Switzerland

**DOI:** 10.3390/ijerph19020865

**Published:** 2022-01-13

**Authors:** Benedikt Gasser, Joel Stouder

**Affiliations:** Departement fuer Sport, Bewegung und Gesundheit, University of Basel, 4001 Basel, Switzerland; Joel.stouder@stud.unibas.ch

**Keywords:** high-altitude pulmonary edema (HAPE), high-altitude cerebral edema (HACE), high-altitude accidents

## Abstract

Background: Despite a potential high risk of acute mountain sickness (AMS) in the Swiss Alps, there is a lack of analyses concerning its relevance over longer periods. In consequence, the aim of this study is to analyze the prevalence of AMS in comparison to other causes of mountain emergencies in recent years in Switzerland. Material and Methods: Based on the central registry of mountain emergencies of the Swiss Alpine Club (SAC), all cases in the period between 2009 and 2020 were analyzed for AMS including the most severe forms of high-altitude pulmonary edema (HAPE) and high-altitude cerebral edema (HACE). Emergencies were assessed for the severity of the event with a National Advisory Committee for Aeronautics (NACA) score. Results: From a total of 4596 high-altitude mountaineering emergencies identified in the observational period, a total number of 352 cases of illnesses were detected. Detailed analysis revealed 85 cases of AMS, 5 cases of HAPE, and 1 case of HACE. The average altitude was 3845 ± 540 m. Most cases were in the canton of Valais, especially in the Monte Rosa region and the mountains of the Mischabel group (Täschhorn, Dom, Südlenz, Nadelhorn, Hohberghorn). There were only three deaths related to high-altitude illnesses; all the other events could be identified as moderate to severe but not life-threatening. Discussion: An emergency due to AMS that requires rescue is unlikely in the Swiss Alps. This does not imply that AMS is not a concern. However, the facts that the maximal altitude is relatively low and that fast self-descents often seem possible probably minimize the likelihood that mountaineers with symptoms contact emergency services.

## 1. Introduction

Switzerland is well known for its beautiful mountains. The high alps in the southeastern and western parts reach up to 4500 m. The Mischabel and Monte Rosa areas are particularly well known for their high mountains. In addition to famous peaks such as Dufourspitze (4634 m), Täschhorn (4491 m), Dom (4545 m), and Matterhorn (4448 m), Switzerland has many smaller peaks with altitudes between 3000 and 4000 m. Switzerland also has many cabins located between 2500 and 3500 m [1,2]. Since an estimated 150,000 people are active in the Swiss Alps in high-altitude mountaineering each year, numerous alpinists there should be at risk of developing the severest forms of high-altitude sickness including high-altitude cerebral edema (HACE) and high-altitude pulmonary edema (HAPE) [1,2,3,4,5]. In principle, people who do not live at high altitude and rapidly ascend to altitudes above 2500 m may develop one or more unpleasant symptoms such as headache, anorexia, and insomnia [3,6]. If several of these symptoms, which may progressively include vomiting and severe headache, are present, the syndrome is defined as acute mountain sickness (AMS), and physical examination of these patients may also disclose tachypnoea, pulmonary rales, and periorbital or peripheral oedema [3]. Data on the incidence and prevalence of these syndromes have been reported for trekkers in Nepal [7,8], Indian soldiers [9], and in places in North America [10,11,12]. Furthermore, an encompassing analysis exists of the prevalence of acute mountain sickness in the Eastern Alps, which detected an overall prevalence rate of 16.2% for AMS, while the prevalence of AMS increased significantly with altitude (2200 m: 6.9%; 2500 m: 9.1%; 2800 m: 17.4%; 3500 m: 38.0%) [13]. Maggiorini and colleagues directly questioned alpinists in SAC huts and examined them clinically [3]. Three of the four huts were in the Bernese Alps: the Konkordiahütte (at 2850 m), the Finsteraarhornhütte (at 3050 m) and the Mönchsjochhütte (at 3650 m). The hut at the highest altitude was the Capanna Margherita (at 4559 m) in the Monta Rosa area in the canton Valais [3]. The general prevalence of AMS was 9% at 2850 m, 13% at 3050 m, 34% at 3650 m, and 53% at 4560 m [3]. In principle, these rates seem valid based on general experience, and the clinical examinations were properly performed. Newer estimations find a normal incidence rate of 0.1 to 15% one to two days after arriving at high altitude depending on factors such as maximum altitude, ascent rate, physical exhaustion, individual susceptibility, and coexisting cardiopulmonary diseases [13,14,15]. Based on the potentially high prevalence, a lot of research has been conducted on pathophysiology in Switzerland, especially in the Monte Rosa region, and preventive measures were developed [16,17,18,19]. Although the Alps are visited each year by millions of tourists and there are approximately 350,000 overnight stays in the huts belonging to the SAC, the likelihood of developing AMS seems low compared to the likelihood of other mountain emergencies [1,2]. However, there is little available information about actual prevalence rates or how often emergency services must deal with AMS. This study aims to answer the following questions: What is the likelihood of requiring professional emergency services for high-altitude sickness in the Swiss Alps? How do the results compare to requiring professional emergency services for other mountain emergencies and how has this changed in recent years?

## 2. Material and Methods

### 2.1. Analyzed Population

All emergency cases related to high-altitude mountaineering in the SAC central registry from 2009 to 2021 were analyzed. The central registry contains data from the Swiss Air Rescue Service (REGA), Air Glaciers Lauterbrunnen, Air Glaciers Sanenland, the Register SAC, the Kantonale Walliser Rettungsorganisation (KWRO), the Snow and Avalanche Research Institute Davos, and the cantonal police registries. The term mountain emergency covers all events where mountaineers require the help of mountain rescue services or are affected by subjective and objective mountain hazards [20,21]. This also applies to illnesses and evacuations of uninjured mountaineers. Each mountain emergency includes the emergency number used, date, rescue organization, event, place, canton, activity, National Advisory Committee for Aeronautics (NACA) score (Table 1), nationality, birth date, sex, place of residence, coordinates, and a case report [22,23]. The case reports were analyzed to determine the occurrence of AMS, HAPE, and HACE. To help identify cases, the subjective version of the Lake Louise score was used (headache plus one additional symptom such as gastrointestinal symptoms, fatigue, weakness, dizziness, lightheadedness [6]). It has to be mentioned that, by definition, at least moderate headache and one of the additional symptoms are necessary to reach the score level of three necessary for diagnosis. However, a sort of uncertainty remains as sometimes only headache was reported but not the severity level. Study analyses were performed in line with the Declaration of Helsinki and its later amendments. Confirmation and a waiver concerning secondary data analysis were received from the local ethics commission.

### 2.2. Data Preparation

In a first step, the causes of mountain emergencies were classified into the following categories: falls, being stuck (unable to go further or back), illness, lightning, crevasse accidents, avalanches, stone falls, ice falls (serac), being lost, material failure, and other. This classification was originally developed by the SAC to enable comparisons of all the disciplines of mountaineering, such as hiking, backcountry skiing, climbing, and classic mountaineering. The classification scheme was unique, meaning that multiple classifications were not allowed. This was followed by a detailed data analysis for the missing entries. Since missing data for fewer than 5% of the entries hardly affected the validity of statements (for example, fewer than 5% of entries were missing values for age), an easily applicable substitution method (mean-value imputation) could be used for further statistical analyses [24,25].

### 2.3. Statistical Analyses

Descriptive statistics were calculated per calendar year for age and NACA scores for the subclass of illnesses. As the hypothesis of normal distributions of the two variables age and NACA score could not be rejected with the Jarque–Bera test, two-sided heteroscedastic t-tests were performed to detect potential sex differences [26,27]. To analyze changes over the observation period, linear regressions of the degree of determination (R^2^) were calculated. Calculations were made with Microsoft Excel (Microsoft Inc., Redmond, WA, USA) and SPSS (Armonk, New York, NY, USA).

## 3. Results

First, a rough analyses revealed 4596 cases in the observational period of 2009–2020; 1028 (22.4%) were female and 3568 (77.6%) were male. There were 1951 (42.4%) cases of being stuck (not able to go further or back), 1348 (29.3%) cases of falls, 352 (7.7%) cases of illnesses, 275 cases of being lost (6%), 266 (5.8%) with stone falls, 162 (3.5%) cases involving crevasse accidents 162 (3.5%), 45 (1%) cases involving avalanches, and in 197 cases (4.3%), the cause was not defined or had an uncommon cause such as lightning or rope-related incidences. A slight increase in cases with time was identified (number of cases = 0.839 × time + 23.9, R^2^ = 0.168). All cases of illnesses were then analyzed in detail. Analyses of the case reports were performed with the help of Lake Louise scores. This analysis showed that 92 of the 352 illness cases (26.6%) had AMS, HAPE, or HACE. Of these cases, 25 (27.1%) were female and 67 (72.8%) were male. Concerning the remaining approximately 260 cases with an illness, diverse reasons were identified, including eye problems due to sunlight (snow blindness), muscle cramps, allergic reactions, hypoglycemia in persistent diabetes, abdominal cramps, asthma, circulatory problems, heart problems, discus hernias, syncope, food poisoning, dehydration, vomiting, and renal colic.

From the case reports, only five cases exhibited clear signs of HAPE, and only one showed clear signs of HACE. Overall, the average age of female alpinists with AMS, HAPE, or HACE was 44.2 ± 12.2 years, and that of male alpinists was 43.1 ± 12.9 years. No statistical difference was detected (*p* = 0.765). Thirty cases were individuals from Switzerland (2 with HAPE), 15 were from Germany (1 with HAPE, 1 with HACE), 9 from Italy, 7 from Great Britain (1 with HAPE), 6 from France, 4 from Slovakia, 2 from Poland, 2 from Austria, and one individual from each of the following countries: Ireland, Canada, Hungary, Russia, the Czech Republic, the Netherlands, Greece, Latvia, Spain, Slovenia, and China (1 with HAPE). The three fatal cases were from Switzerland, Latvia, and Slovakia.

**Figure 1:**
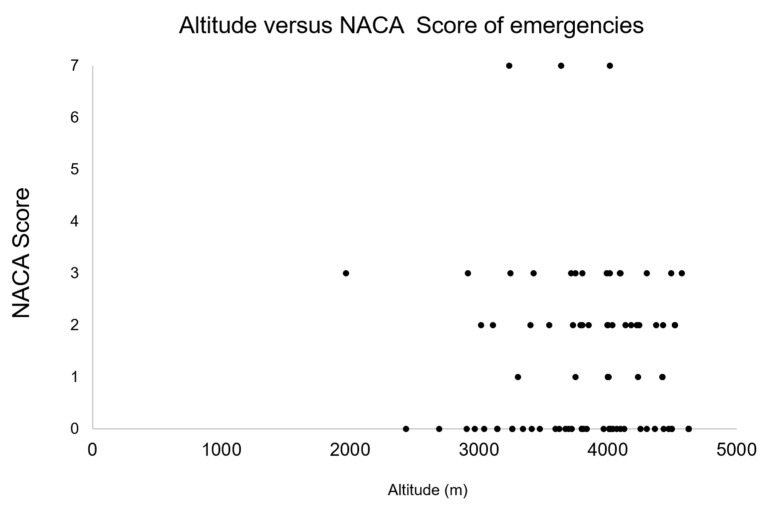
Distribution of altitudes of the events (*x*-axis) and NACA scores (*y*-axis). Mean NACA score was 1.82 ± 1.59, and mean altitude was 3845 ± 540 m. Only three deaths were found (NACA score = 7); for all other events, NACA score was 3 or less.

**Figure 2:**
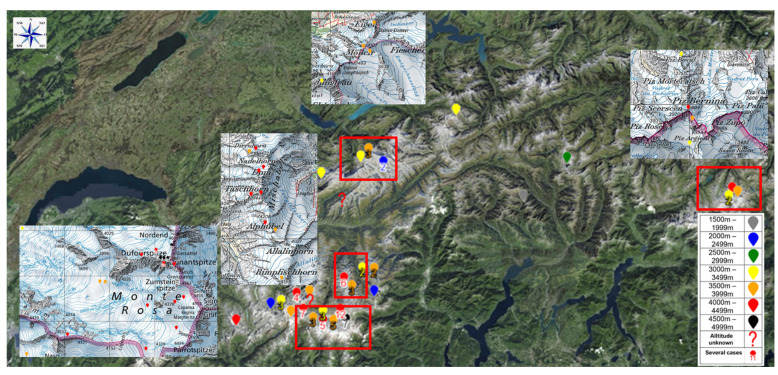
A map of Switzerland with locations of the cases (color-coded dots show the altitude of the event with the number of events if several). Four central regions could be identified: in the Bernese alps in the Jungfrau area, in the eastern part of the Bernina area, in the Mischabel area and in the Monte Rosa region.

The average altitude at which victims were rescued was 3845 ± 540 m, and the average NACA score was 1.82 ± 1.59 (Figure 1). For female alpinists, the average NACA score was 1.72 ± 1.25; for males, it was 1.86 ± 1.72. No significant difference was detected (*p* = 0.686). Concerning location, four main areas could be identified: Monte Rosa (Valais), Mischabel (Valais), Jungfrau (Bern), and Bernina (Grisons) (Figure 2).

## 4. Discussion

The aim of this study was to assess the prevalence of specific altitude illnesses in mountaineers rescued in the Swiss Alps. Some factors limit the probability of developing AMS in the Alps. First, the emergency system is well established and fast rescue is often possible. Second, alpinists usually do not develop severe forms of AMS and can go down by themselves to moderate altitudes since the distances are normally relatively short. Compared to the Himalayas and the Andes, it is often possible to return from high-altitude terrain very fast, maybe even with the help of a cable car. Third, the altitude is relatively low compared to the Himalayas or Andes, so in the highest areas, the partial oxygen pressure is still only reduced to approximately fifty percent compared to sea level [16,17,18,19,28,29]. Nevertheless, the SAC central registry was analyzed from 2009 to 2020. Illnesses were less common than other causes in the central registry, such as falls or being stuck. Approximately one-quarter of subjects affected by an illness had AMS, HAPE or HACE. This was approximately 2% of all registered cases in the sample. The mean NACA score was below 2, which indicates a moderate to severe but not life-threatening case. AMS was mainly found in the Valais mountains, especially in the Monte Rosa massif and the mountains of the Mischabel group (Täschhorn, Dom, Südlenz, Nadelhorn, Hohberghorn). In relation to the entire observational period (2009–2020), the mortality rate was low, with only 3 deaths in 12 years. In comparison, the mountain emergency statistics for the last 5 years show an average mortality rate of approximately 21.8 deaths per year while high-altitude mountaineering [30]. According to the report, they all had a severe form of AMS according to Lake Louise score. The low mortality rate would underpin the claim that AMS is a rare event in the Swiss Alps. Except for the three deaths, all the other cases had a NACA score of 3 or less (Figure 1). Focusing on further aspects, Maggiorini et al. (1990) and Hackett et al. (1976) found that AMS was evenly distributed among both sexes, which is in line with the findings here. More men (70.9%) were affected compared to woman. However, the share is more or less the same as from the total analysis (78.1%). A priori AMS seems a rare phenomenon in the Swiss Alps, especially when one considers the claim of the SAC that 150,000 alpinists are active each year in Switzerland at high altitude, which yields a prevalence of only 0.048% (less than one case per 10,000) [2,30,31]. Several reasons for a low prevalence requiring emergency treatment can be identified. This analysis only included cases that resulted in a response by emergency services such as Air Glacier or REGA. Cases of self-assignment, for example, to an emergency department or a General Practitioner were thus not captured in the statistics. Additionally, many mountaineers affected with signs or symptoms of AMS probably simply started to descend on their own.

Although they were beginning to show signs of AMS, even some cases with signs of HAPE or HACE, they are not recorded in the statistics. Descents of this manner are often easily possible in the Swiss Alps, as the distances to moderate altitude are often short. In addition, there are often transport options such as cable cars for returning to lower altitude levels at a high speed. A further limitation was the accuracy of the case reports, since emergency services were probably happy to have successfully performed a rescue and were not focused on commenting on the cases in detail. To summarize, emergencies due to AMS, HACE or HAPE exist but are rare in the Swiss Alps.

## 5. Conclusions

Emergencies due to AMS, HACE or HAPE exist in the Swiss Alps, but they should be considered rare events. Most cases were detected in the Valais in the Monte Rosa region (Castor, Pollux, Lyskamm, Signalkuppe, Parrotspitze, Zumsteinspitze, and Dufourspitze) or in the Mischabel group (Täschhorn, Dom, Südlenz, Nadelhorn, and Hoberghorn). There are several reasons for the low prevalence requiring emergency treatment of AMS, such as the relatively low maximum height of the Alps (compared to the Himalayas or the Andes). Furthermore, it is often possible to reach high-altitude areas with the help of a cable car, for example, so quick descent is also possible, which is definitely less common in the Andes or the Himalayas. AMS can occur within 24 h of ascent, particularly with rapid ascent and/or exertion [6]. Lake Louise scoring criteria suggests 6 h as a minimum exposure duration before measurement but acknowledges that symptoms can occur prior to this [6]. Yet, most forms are mild, perhaps because many peaks are often quickly reached (within 24 h) and can be completed in one day, so a severe form of AMS cannot develop.

## Figures and Tables

**Table 1:** National Advisory Committee for Aeronautics (NACA) Score [24,25].

NACA 0	No injury or disease.
NACA I	Minor disturbance. No medical intervention is required. e.g., slight abrasion.
NACA II	Slight to moderate disturbance. Outpatient medical investigation, but usually no emergency medical measures necessary. e.g., fracture of a finger bone, moderate cuts, dehydration.
NACA III	Moderate to severe but not life-threatening disorder. Stationary treatment required, often emergency medical measures on the site. e.g., femur fracture, milder stroke, smoke inhalation.
NACA IV	Serious incident where rapid development into a life-threatening condition can not be excluded. In the majority of cases, emergency medical care is required. e.g., vertebral injury with neurological deficit, severe asthma attack; drug poisoning.
NACA V	Acute danger. e.g., third grade skull or brain trauma, severe heart attack.
NACA VI	Respiratory and or cardiac arrest
NACA VII	Death

## Data Availability

For reasons of personal rights of victims data is in principle not accessible.
